# Application of singular value decomposition analysis to time-dependent powder diffraction data of an *in-situ* photodimerization reaction

**DOI:** 10.1107/S1600577514004366

**Published:** 2014-04-02

**Authors:** Ahmed F. Mabied, Shunsuke Nozawa, Manabu Hoshino, Ayana Tomita, Tokushi Sato, Shin-ichi Adachi

**Affiliations:** a X-ray Crystallography Laboratory, Solid State Department, Physics Division, National Research Center, El Buhouth (El-Tahrir) Street, Dokki, Giza 12622, Egypt; b Photon Factory, High Energy Accelerator Research Organization, KEK, Tsukuba 305-0801, Japan; c Department of Chemistry and Materials Science, Tokyo Institute of Technology, Meguro-ku, Japan; d CREST, Japan Science and Technology Agency, Japan; e Department of Materials Structure Science, School of High Energy Accelerator Science, KEK, The Graduate University for Advanced Studies, Sokendai, Tsukuba 305-0801, Japan; f PRESTO, Japan Science and Technology Agency, Japan

**Keywords:** singular value decomposition, anthracene derivatives, photodimerization, time-dependent, X-ray powder diffraction

## Abstract

Singular value decomposition (SVD) analysis has important applications for time-dependent crystallographic data, extracting significant information. Herein, a successful application of SVD analysis of time-resolved powder diffraction data over the course of an *in-situ* photodimerization reaction of anthracene derivatives is introduced. SVD revealed significant results in the case of 9-methylanthracene and 1-chloroanthracene. The results support the formation of the 9-methylanthracene stable dimer phase and suggest the existence of an excimer state.

## Abstract 2

A successful application of singular value decomposition analysis to time-dependent powder diffraction data of an *in-situ* photodimerization reaction of some anthracene derivatives is presented.

## 1. Introduction   

The rapid development of time-resolved X-ray experiments has raised challenges regarding data analysis and the extraction of significant information. Singular value decomposition (SVD) analysis introduces an excellent solution, where large data sets containing a number of values can be reduced to fewer data sets containing significant values while conserving the correlation of variables of the original data. SVD is able to decompose time-resolved data into time-independent and time-dependent components. Also, it can help to extract meaningful signals from noisy data (Henry & Hofrichter, 1992 ▶; Rajagopal *et al.*, 2004 ▶).

SVD analysis has had a variety of successful applications in crystallography; for instance, with time-resolved small-angle X-ray scattering (Okamoto & Sakurai, 2003 ▶) and time-resolved macromolecular crystallographic data (Zhao & Schmidt, 2009 ▶; Rajagopal *et al.*, 2004 ▶). In addition, it has been successful in many other areas such as spectroscopy (Berlich *et al.*, 2005 ▶; Henry & Hofrichter, 1992 ▶; Henry, 1997 ▶), gene expression data (Alter *et al.*, 2000 ▶; Wall *et al.*, 2003 ▶), scientific computing and signal processing (Lahabar & Narayanan, 2009 ▶). Among these applications, SVD has proved efficient in treating time-dependent crystallographic and spectroscopic data. It can reveal important information by extracting small changes from the data that could not be detected directly (Oka *et al.*, 2000 ▶; Rajagopal *et al.*, 2004 ▶; Zhao & Schmidt, 2009 ▶; Henry & Hofrichter, 1992 ▶).

The present study sheds some light on the advantages of applying SVD analysis to time-resolved crystallographic data in the case of powder diffraction. The target here is to treat the data of *in-situ* photodimerization time-dependent powder diffraction measurements of the anthracene derivatives 1-chloroanthracene (1-chA) and 9-methylanthracene (9-MA).

Upon illumination with light of wavelength >300 nm, anthracene derivatives undergo important photodimerization to form a stable dimer phase, which can be dissociated into its initial monomers either thermally or by illumination under light of wavelength <300 nm (Bouas-Laurent *et al.*, 2001 ▶; Schmidt, 1971 ▶). Based on their photodimerization properties, many applications have been developed, such as the fabrication of photo-switchable devices (Zhao *et al.*, 2008 ▶), control of biological reactions (Molard *et al.*, 2006 ▶) and with optical storage memory devices (Dvornikov & Rentzepis, 1996 ▶).

Reported possible processes during monomer–dimer transformations due to the photoreaction are as follows: monomers absorb the light (2M + *h*ν → M*) and then relax to instantly excited monomers (M^ex^, excimer). The excimer could either disintegrate into stable monomers (M^ex^ → 2M + *h*ν) or form a stable dimer (M^ex^ → D). However, forming the excimer is faster than forming a stable dimer (Takegoshi *et al.*, 1998 ▶; Ferguson & Mau, 1974 ▶). Moreover, Aladekomo (1973 ▶) reported that 9-MA is one of the unique materials which enables the study of excimer and stable dimer.

**Figure 1:**
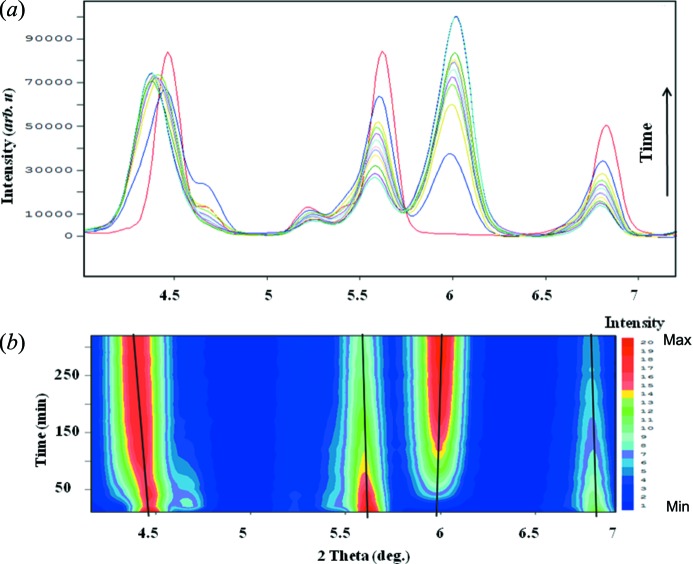
Time-resolved powder diffraction profiles of 9-MA photodimerization showing the deviations with 2θ. (*a*) Raw data. (*b*) Two-dimensional contour plot.

In our previously reported photodimerization study of 9-MA (Mabied *et al.*, 2012 ▶), the time-dependent powder diffraction data of 9-MA monomer and dimer phases were analyzed quantitatively based on the averaged structure in each phase. The study overcomes the drawbacks of photoreactions in a single crystal by the application of powder diffraction reaching higher completion (63.8%) of the dimer fraction. The suggested phase-transition kinetics reveal that small parts of the reactant phase (nuclei of the dimer) appear randomly in the parent phase at the beginning of the reaction. This means that the dimer and its associated excimer state should exist. In addition, it could enhance the change of the averaged diffraction intensity in the monomer phase despite the dimer peaks not yet having appeared, as shown in Figs. 1 ▶ and 2 ▶. Therefore, the aim of this study is to find any traces of the excimer state by applying SVD analysis, which could not be observed directly using Rietveld refinement.

## 2. Experimental   

1-Chloroanthracene powder (95% pure) was obtained from Sigma Aldrich Company and 9-methylanthracene (98% pure) was supplied by Wako Chemical Co., Japan. All the samples were ground using an agate mortar and pestle after recrystallization from acetone and hexane solvents. The samples were filled into borosilicate glass capillaries of diameter 0.4 mm (Hilgenberg, Germany).

The photodimerization experiment was carried out *in-situ* at the X-ray time-resolved beamline NW14A of the Photon Factory Advanced Ring (PF-AR) facility, KEK, Japan. Details of the beamline are given elsewhere (Nozawa *et al.*, 2007 ▶). The X-ray beam was set to an energy of 18.0 keV (λ = 0.689 Å). The samples were mounted on a horizontal ϕ-axis and rotated around this axis for better counting statistics. The sample-to-detector distance was set as 150 mm and calibrated using the 111 diffraction ring from standard silicone powder. Powder diffraction rings were collected at ambient temperature, which was about 302 K at the sample position, using a Mar165 CCD detector. Visible light from a xenon lamp with a visible mirror module (MAX-301; 385–740 nm, 300 W; Asahi Spectra) illuminated the sample undergoing photodimerization during data collection. IR spectra were measured on a Shimadzu IR Prestige-21 FTIR spectrometer by dispersing samples in KBr pellets. UV–Vis absorption spectra were recorded using a Varian Cary 50 Conc spectrophotometer in a cyclohexane solution.

## 3. SVD and data treatment   

### 3.1. SVD   

Mathematically, singular value decomposition can be defined as follows. An *m*-by-*n* real matrix *A* (*m* ≥ *n*) can be decomposed into three matrices *U*, *S* and *V* ^T^ [*A*_*m*,*n*_ = *USV* ^T^, equation (1)]. *U* is an *m*-by-*n* (*m* ≥ *n*) matrix having the property that *U* ^T^ *U* = *I*_*n*_, where *I*_*n*_ is the identity matrix; columns of *U* are called the left singular vectors of *A*. The matrix *S* is an *n*-by-*n* diagonal matrix with non-zero elements in descending order (*s*_1_ ≥ *s*_2_ ≥…≥ *s*_*n*_ ≥ 0) and called the singular values of *A*, which also indicates the matrix rank. *V* ^T^ is the transpose of an *n*-by-*n* matrix *V* (*V* ^T^ *V* = *I*_*n*_), where *I*_*n*_ is the identity matrix; columns of *V* are called the right singular vectors of *A*. Further details can be found elsewhere (Henry & Hofrichter, 1992 ▶; Stoer & Bulirsch, 2002 ▶; Unonius & Paatero, 1990 ▶; Golub & Van Loan, 1996 ▶).
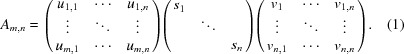
Physically, according to the literature (Henry & Hofrichter, 1992 ▶; Unonius & Paatero, 1990 ▶), the SVD method can factorize an experimental data *m*-by-*n* matrix into several components matrices. The columns of the *U* matrix represent the measurements base spectrum of the original data and the *S* elements give its singular values, which indicate the importance of the *U* spectrum. The singular values are arranged in descending order according to their magnitudes. The *V* matrix gives the associated time-dependent vectors of the *U* elements. In other words, based on the obtained singular values (*s*_1_,…, *s*_*n*_) and the characteristics of the *U* spectrum, the importance of the *U* columns (*u*_1_,…, *u*_*n*_) is decided, and hence the associated time-dependent vectors of *V* (*v*_1_,…, *v*_*n*_) can be fitted (Henry & Hofrichter, 1992 ▶; Unonius & Paatero, 1990 ▶). The SVD results can be interpreted successfully based on global fitting of the column vectors of the matrix *V* extraction of time-independent correlations (Zhang *et al.*, 2004 ▶; Van Wilderen *et al.*, 2011 ▶; Henry & Hofrichter, 1992 ▶; Unonius & Paatero, 1990 ▶). Visualization of the singular values is the most important step for understanding the results of the *U*, *S* and *V* matrices and to decide the meaningful components. One of the graphical methods used is a one-dimensional plot, where the height of any one singular value is indicative of its importance in explaining the data. The relative variances [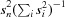] are often plotted, where the square of each singular value is proportional to the variance explained by each singular vector. These kinds of plots are called scree plots, as referred to by Cattell (Cattell, 1966 ▶; Wall *et al.*, 2003 ▶).

### 3.2. Data treatment   

The standard 2θ *versus* intensity powder diffraction patterns through the measurement time were obtained from the recorded powder diffraction rings using *Fit2D* (Hammersley *et al.*, 1996 ▶). The geometrical correction was applied and the beam center shadows and contamination spots were masked (Hammersley *et al.*, 1996 ▶). For more accuracy, the background was subtracted from the data using *Powder3D* software (Hinrichsen *et al.*, 2006 ▶) before SVD analysis. *WinPLOTR* (Roisnel & Rodriguez-Carvajal, 2001 ▶) and *Powder3D* (Hinrichsen *et al.*, 2006 ▶) were used to visualize the time-resolved data.^1^

**Figure 3:**
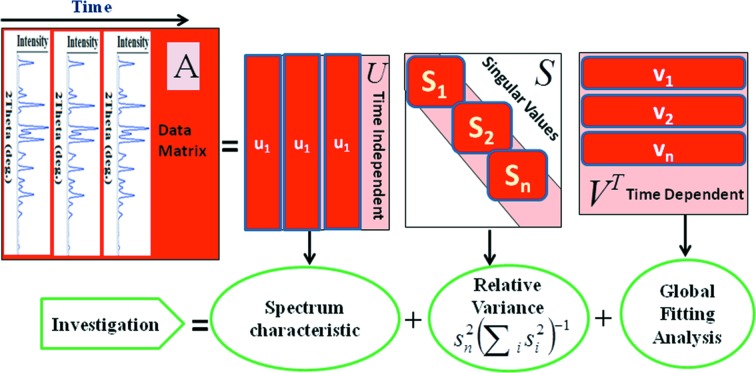
Schematic diagram showing how SVD works with time-resolved X-ray powder diffraction data.

The corrected powder data were set into an *m*-by-*n* matrix, where the columns are the diffraction intensity at the 2θ angle points (Fig. 3 ▶) [for further reading about data treatment, see Henry & Hofrichter (1992) ▶ and Oka *et al.* (2000) ▶].

The SVD was computed according to the reported procedures, where the data matrix *A* can be described by an *m*-by-*n* matrix *P*(2θ) and an *n*-by-*n* matrix *C*(*t*) as *A*(*s*, *t*) = *P*(2θ)*C*(*t*) = *USVT*. Here, *P*(2θ) and *C*(*t*) represent the diffraction pattern of the independent components and their concentrations as a function of time *t*, respectively. As mentioned above, *U* contains the basis spectra of the diffraction pattern, *S* contains the associated eigenvalues, and *V* contains the time-dependence of the basis spectra.

The statistical weights for the data set **A** have been considered. The weight matrix *W* is defined as *W*_*ij*_ = 1/σ_*ij*_ (*i* = *j*) and *W*_*ij*_ = 0 (*i* ≠ *j*), where the error of the data element *A* (2θ_*i*_,*t*_*j*_) is described as σ_*ij*_. Since the measurement time is identical for all data, the error would depend on 2θ. The value of σ_*ij*_ is given as the average of the square root of *A*_*ij*_ for all frames (*j* = 1 to *n*). Therefore, the final data set to be analyzed should be *WA* = *USVT* = *WU′SVT*, where *U*′ = *B* ^−1^ *U*. Then, *A* = *U*′*SV* ^T^.

The matrix dimensions were (1233, 59) and (1095, 42) for 9-MA and 1-chA, respectively. According to (*A* = *U*′*SV* ^T^), the SVD was calculated and produced the three matrices *U*, *S* and *V*. The results (see §4) of the time-dependent spectra of the *V* matrix were plotted. Fitting analysis of the *V* curves showed the best fit function for the double exponential function [equation (2)]. As the results do not correspond directly to independent states in the photoreaction, the diffraction profiles need to be reconstituted. Global fitting analysis can extract the time-independent correlations and their associated rate constants. Therefore, it was applied to the 9-MA data. The parameters of equation (2) have been obtained successfully and can be defined as follows: *A*_0_ is a time-independent part, which should be unchanged during the photoreaction. *A*_1_ and *A*_2_ are the changing parts; their rate constants are *k*_1_ and *k*_2_, respectively. The powder diffraction profile during the photoreaction can be considered as a summation of all these profiles *A*_0_, *A*_1_ and *A*_2_. All SVD calculations and fitting were performed using *IGORPro* (Wave Metrics, 2011 ▶),
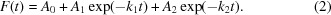



## 4. Results and discussion   

SVD analysis of the present data gives the three matrices *U*, *S* and *V*. Investigation of the results can be considered as an aggregate of three major steps (Fig. 3 ▶): the first is determining the important singular values from the *S* matrix using a visualization method (such as relative variance); the second is checking the associated base spectrum characteristics of the matrix *U*; and the third is examining the behaviour of the time-dependent vectors of the matrix *V* using global fitting analysis.

**Figure 4:**
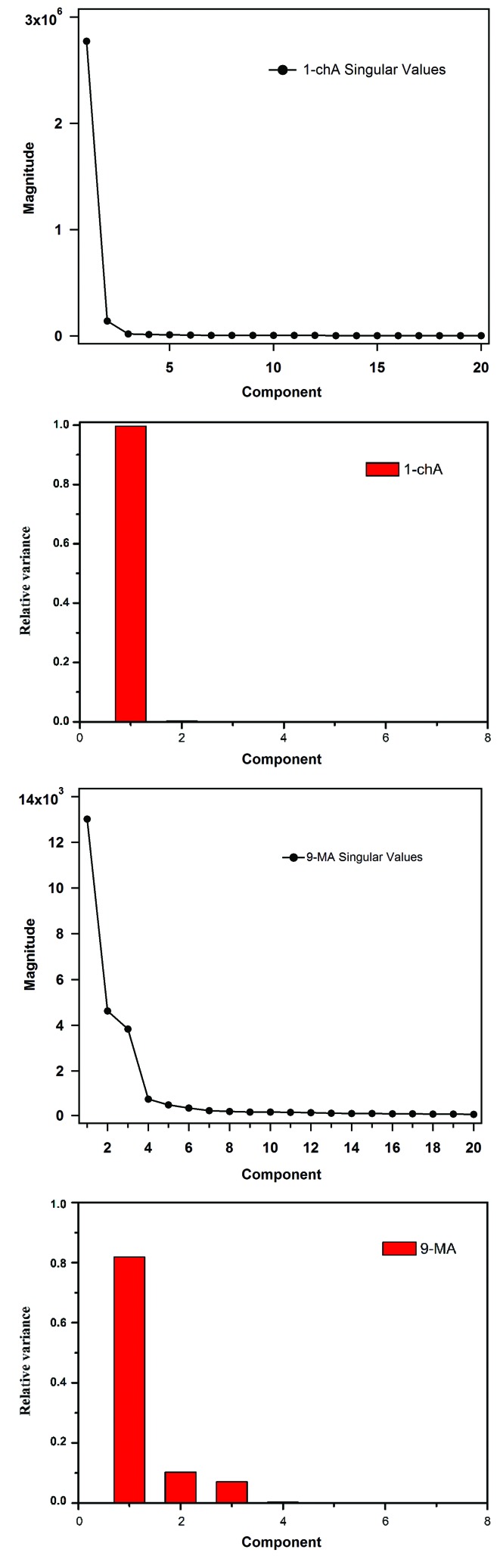
Comparison of the resultant singular values of SVD analysis of time-resolved powder diffraction data of both 9-MA and 1-chA data, illustrating the singular values *versus* their components of the obtained *S* matrix and the relative variance plot (red bars) showing the importance of each component.

Fig. 4 ▶ shows a comparison of the resultant singular values of the 9-MA and 1-chA data. It illustrates the singular values *versus* their data components of the obtained *S* matrix. The importance of every component is indicated by the relative variance plot (red bars). The significance was decided based on the visualized singular values and their distinctive *U* and *V* spectra.

**Figure 5:**
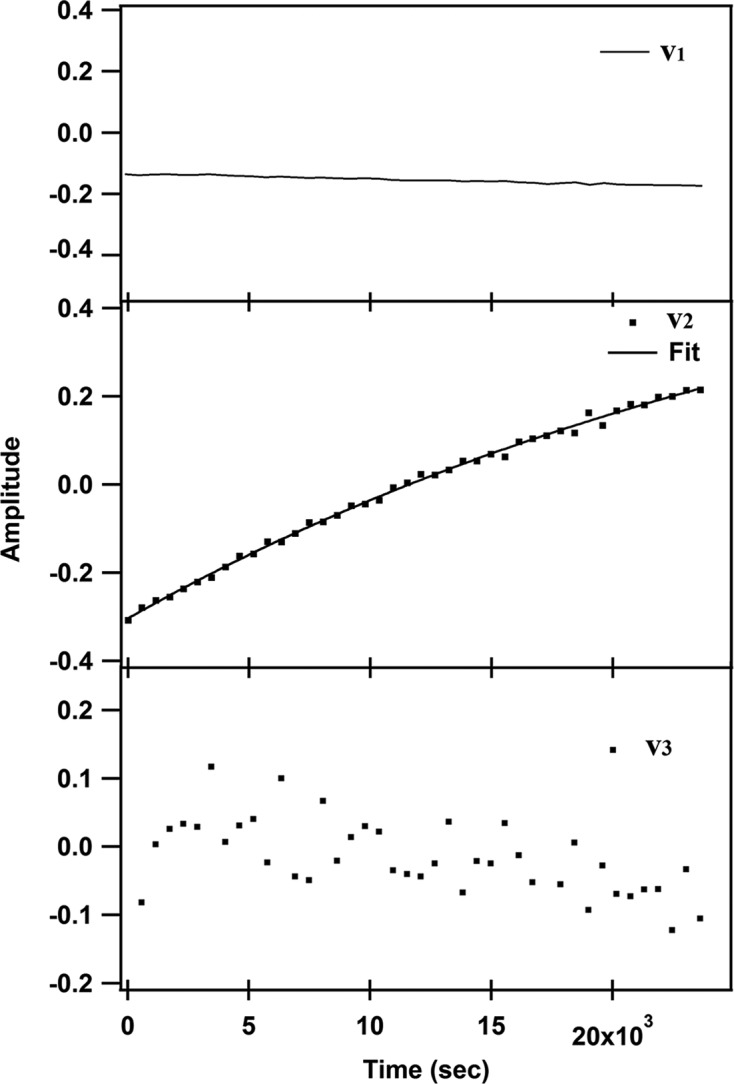
The first three significant amplitude vectors of *V*, associated with the singular values obtained from SVD analysis of 1-chA data.

**Figure 6:**
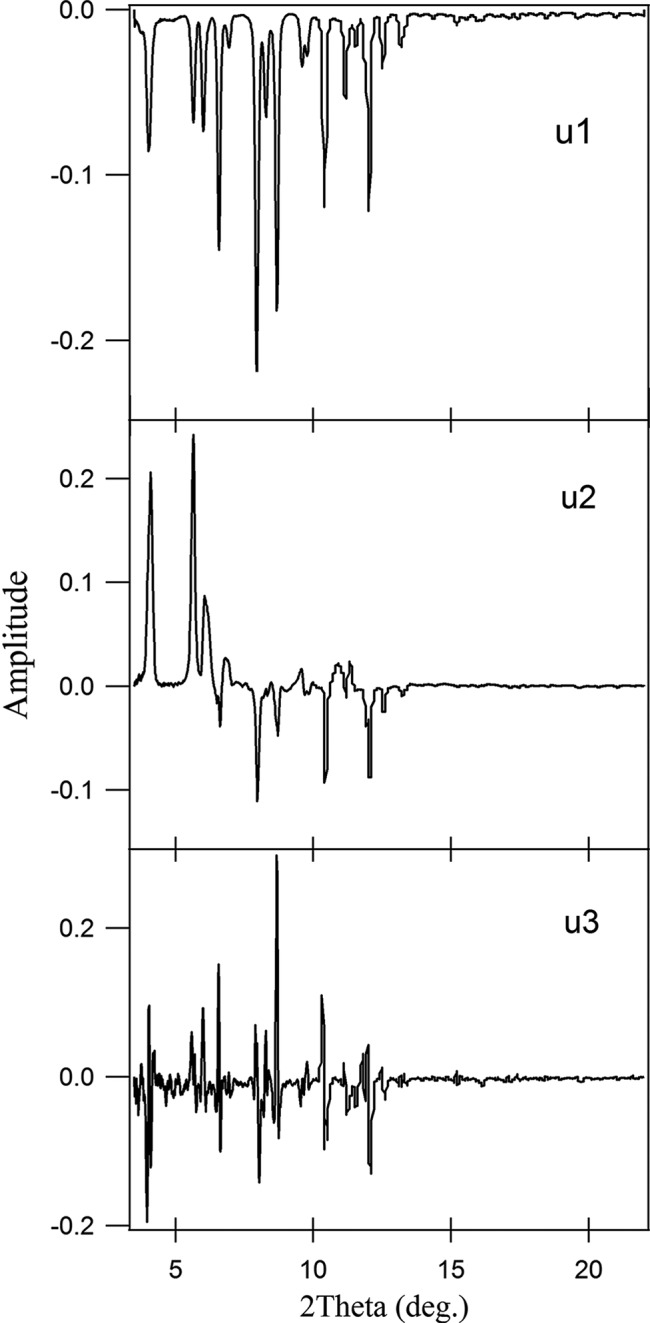
*U* spectra (*u*_1_, *u*_2_ and *u*_3_) associated with the first three singular values of 1-chA.

For 1-chA data, there is a distinguishable singular value component (*s*_1_ = 2.8 × 10^6^) and relatively smaller *s*_2_ component, as shown in Fig. 4 ▶. The *s*_1_-related *v*_1_ spectrum shows almost time-independency (Fig. 5 ▶) . The associated *u*_1_ spectrum characteristics are similar to the original diffraction pattern of the 1-chA monomer phase (Fig. 6 ▶).

**Figure 7:**
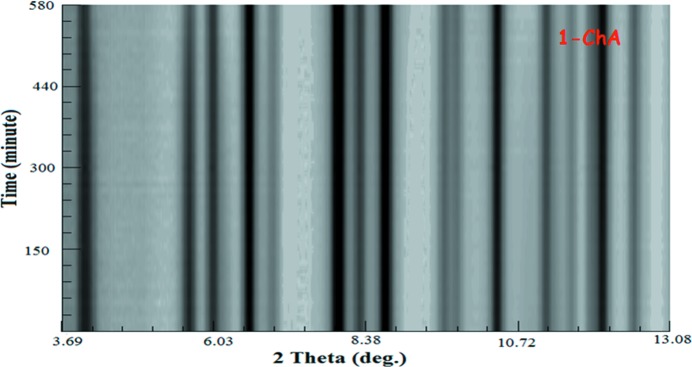
Waterfall presentation of the propagation of typical Bragg diffraction peaks of 1-chA.

**Figure 8:**
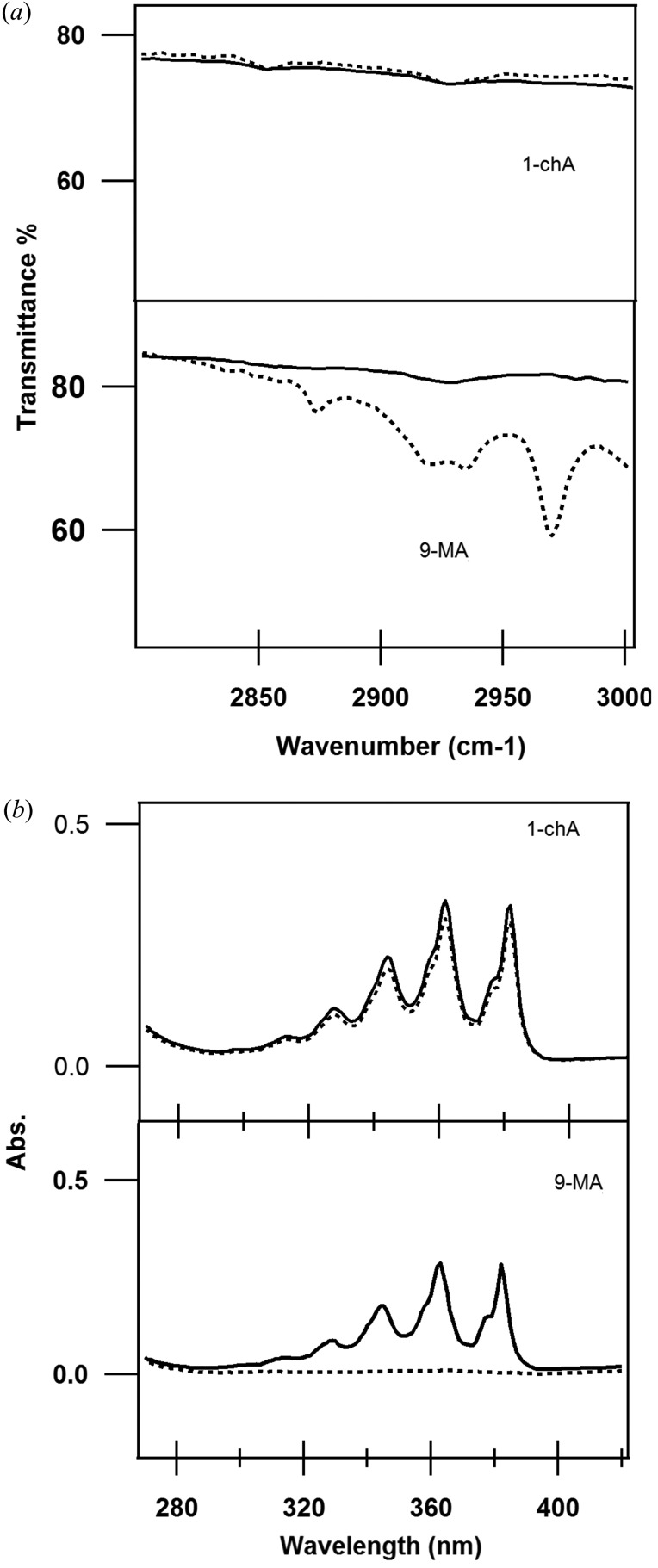
Selected bands of the measured spectra of 9-MA and 1-chA. The dotted lines, representing the illuminated samples, show that there is no change in the case of 1-chA, contrary to 9-MA. (*a*) IR spectra. (*b*) UV–Vis spectra.

Fig. 7 ▶ shows that there is almost no change in the position of the peaks of the 1-chA diffraction pattern during the illumination in contrast to 9-MA (Fig. 1 ▶). This indicates that the photodimerization of 1-chA barely proceeded. The consistency of IR and UV–Vis spectra under the photoirradiation condition (Fig. 8 ▶) ensured less activity of this reaction. However, *v*_2_ showed time-dependency (Fig. 5 ▶): the best curve fitting matched the double exponential function [equation (2)], which gave fast behaviour only with almost the same rate constant (*k*_1_ = 3.148 ± 0.093 × 10^−5^ s^−1^ and *k*_2_ = 3.1 ± 0.093 × 10^−5^ s^−1^) in contrast to the case of 9-MA, as shown in Table 1 ▶. The existence of such time-dependency character, even if the dimer phase does not appear, could suggest that it is coming from the excimer state, which can disintegrate into the stable monomers without forming the dimer phase of 1-chA. This is in agreement with the reported possible paths of the photodimerization reaction as mentioned in the *Introduction* [Sec sec1].

**Figure 9:**
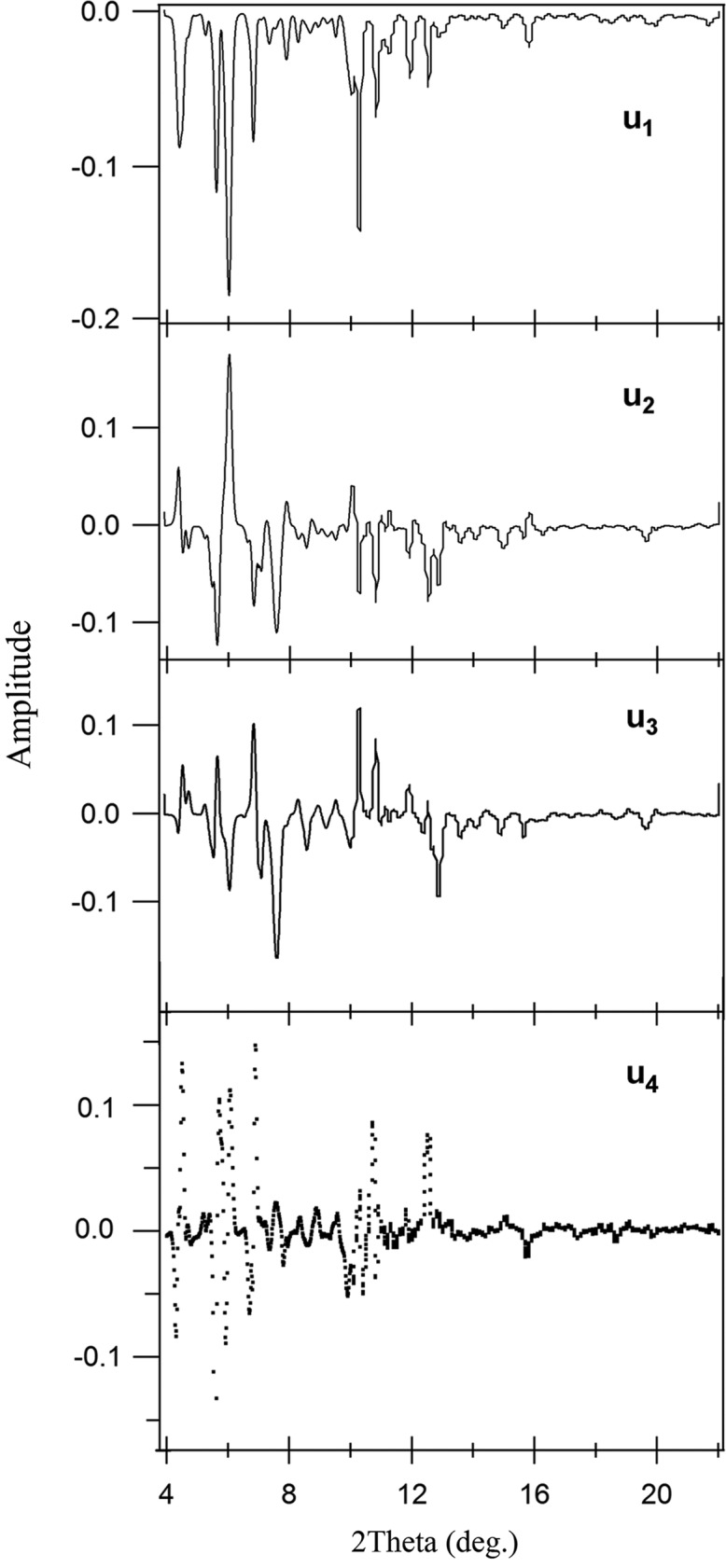
*U* spectra (*u*_1_, *u*_2_, *u*_3_ and *u*_4_) associated with the first four singular values *s*_1_ = 13021.54, *s*_2_ = 4620.65, *s*_3_ = 3835 and *s*_4_ = 762.241 for SVD analysis of the 9-MA data.

**Figure 10:**
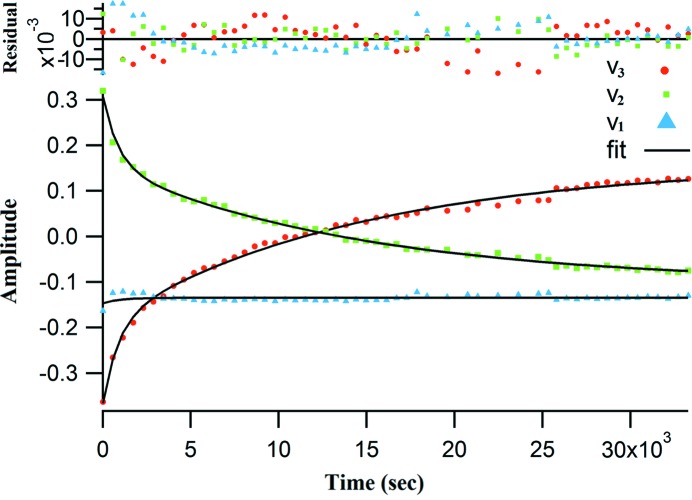
Plot of global fitting results of the time-dependent *V* spectra for 9-MA SVD analysis. A residual plot shows the residuals of *v*_1_, *v*_2_ and *v*_3_ on the vertical axis and the independent variable on the horizontal axis.

The visualization of the 9-MA data singular values (Fig. 4 ▶) indicates that three components are significant. The first three singular values (*s*_1_ = 13021.54, *s*_2_ = 4620.65 and *s*_3_ = 3835) are larger than the other values and give the non-random *U* spectrum (Fig. 9 ▶), which could point out that the higher singular values come from noise. The accompanying *u*_1_, *u*_2_ and *u*_3_ (Fig. 9 ▶) spectra and their time courses *v*_1_, *v*_2_ and *v*_3_ (Fig. 10 ▶) can give information about this significance.

However, the first component (*s*_1_) is the largest one. The distinct *u*_1_ spectrum was similar to the diffraction pattern of the 9-MA dimer phase (Fig. 1 ▶) except for its negative sign (Fig. 9 ▶), which corresponds to the sign of *v*_1_ (Fig. 10 ▶). Oka *et al.* (2000 ▶) have reported similar cases. *v*_1_ showed little time-dependency at the lower values, as noticeable in Fig. 10 ▶; the character of *v*_1_ arises from the existence of the stable dimer. The other two components *u*_2_ and *u*_3_ are also significant because of their distinct *u*_2_ and *u*_3_ spectra, which are distinguishable from noise.

The first *V* spectrum (*v*_1_) was almost time-independent during the measurement, while the second spectrum (*v*_2_) was seen to decay and the third (*v*_3_) to grow, as shown in Fig. 10 ▶. This means that, even if the amount of one component decreased, another component increased to compensate for the decrease in the diffraction intensity. This is consistent with similar cases (Okamoto & Sakurai, 2003 ▶).

The characteristics of the *U* spectrum support this assumption, where the *u*_2_ and *u*_3_ spectra look like a diffraction pattern composed of diffraction peaks of the 9-MA monomer and dimer phases (Figs. 1 ▶ and 9 ▶) while neglecting the peak directions. The associated *v*_2_ and *v*_3_ spectra showed notable time-dependency in contrast to the *v*_1_ spectrum (Fig. 10 ▶). The *v*_2_ and *v*_3_ spectra were fitted successfully with a double exponential function [equation (2)] using global fitting analysis (Fig. 10 ▶). The results of the global fitting analysis are given in Table 1 ▶. For simplicity, it can be imagined roughly that the X-ray diffraction profile is a summation of all three profiles that do not correspond directly to the independent states in the photoreaction, where *A*_0_ is time-independent; *A*_1_ and *A*_2_ are time-dependent components. However, as all of them are describing the same reaction, part *A*_0_ also exhibits little time-dependency especially at the early stages of the reaction.

As given in Table 1 ▶, *A*_1_ and *A*_2_ provide the rate constants *k*_1_ and *k*_2_, showing the fast and slow parts *A*_1_ and *A*_2_, respectively. The presence of such a fast time-dependency character strongly indicates that it is coming from the excimer, which can be disintegrating into the stable dimer phase. This is in agreement with reports on the monomer–dimer transformations due to the photoreaction, where the excimer either decays to stable monomers or forms a dimer phase. Forming the excimer state was found to be faster than forming the stable dimer (Takegoshi *et al.*, 1998 ▶; Ferguson & Mau, 1974 ▶), and it has been reported that the excimer phase is unstable at room temperature (Horiguchi *et al.*, 1987 ▶).

The appearance of the slower component *A*_2_ is also compatible with the reported literature for the mechanism of photodimerization, where the photodimerization mechanism involves a slow process forming a stable dimer in addition to a faster one for the metastable state (excimer) (Birks & Aladekomo, 1963 ▶; Takegoshi *et al.*, 1998 ▶; Ferguson & Mau, 1974 ▶).

A more detailed discussion about photoisomerization kinetics and the phase growth mechanism of 9-MA has been given by Mabied *et al.* (2012 ▶). The dimer peaks were distinguished and could be analyzed using Rietveld refinement after about 30 min from the beginning of the reaction (Fig. 1 ▶). However, the dimer and its associated excimer state should exist at the start of the reaction and could enhance the change of the average diffraction intensity in the monomer phase even though the dimer peaks have not yet appeared. In the present work, SVD has treated all of the time-dependent data sets from the start of the reaction showing traces to the excimer state; such a way of data treatment may also cause the variation of the numerical magnitude of the rate constants between the reported growth rate (Mabied *et al.*, 2012 ▶) and the present study (Table 1 ▶). Similar (4π+4π) photodimerization studies have reported rate constants with similar orders of the numerical results, *k* = 9.7 (14) × 10^−8^ s^−1^ and *k* = 2.1 (26) × 10^−6^ s^−1^ (Cao *et al.*, 2010 ▶).

The UV–Vis and IR spectra supported the SVD analysis results of 9-MA. The IR spectrum showed the appearance of the aliphatic C—H saturated bond for the dimer phase (Fig. 8*a* ▶) below 3000 cm^−1^ (Singh & Sandorfy, 1969 ▶). The absorption peaks between 350 and 400 nm (Fig. 8*b* ▶) dis­appeared in the UV–Vis spectrum of the illuminated 9-MA, which was reported as an indication of the formation of the dimer phase (Tillman *et al.*, 2007 ▶).

## 5. Conclusions   

SVD analysis for time-resolved powder diffraction of photodimerization reactions was introduced in order to effectively extract meaningful parameters from a small amount of changes from the time-dependent crystallographic data. 1-chA and 9-MA were successful examples of SVD application to the analysis of time-dependent powder diffraction experiments.

The results of SVD analysis revealed significant information of 9-MA and 1-chA photodimerization, which strongly suggest the existence of the excimer state even if it is difficult to detect directly, and supported the formation of the 9-MA stable dimer phase.

SVD analysis of time-dependent powder diffraction experiments can be recommended as a powerful tool dis­covering the important features hidden in their data sets, which leads to more useful applications. Using visualization methods, in addition to considering the physical description of the model under study and global fitting, is a very helpful method for investigation of the results.

## Supplementary Material

Raw X-ray diffraction data of both 9-MA and 1-chA before processing, used for SVD analysis. DOI: 10.1107/S1600577514004366/co5048sup1.zip

## Figures and Tables

**Figure 2:**
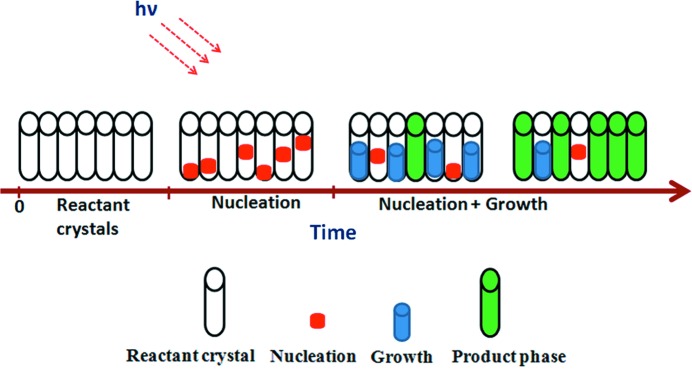
Schematic diagram of the 9-MA solid-state (4π+4π) photodimerization kinetics.

**Table 1:** Global fitting result of 9-MA data showing parameters of equation (2)

	*A*_0_ (constant)	*A*_1_ (*k*_1_ = 6.8 0.3 10^5^s^1^)	*A*_2_ (*k*_2_ = 109.0 090.0 10^5^s^1^)
*v*_1_	0.1357 0.0007		
*v*_2_	0.1604 0.0055	0.3488 0.0047	0.1793 0.0086
*v*_3_	0.1029 0.0042	0.2588 0.0046	0.1520 0.0079

## Footnotes

1 Raw X-ray diffraction data of both 9-MA and 1-chA are available from the IUCr electronic archives (Reference: CO5048).
